# Spinal cord autoregulation using near-infrared spectroscopy under normal, hypovolemic, and post-fluid resuscitation conditions in a swine model: a comparison with cerebral autoregulation

**DOI:** 10.1186/s40560-020-00443-6

**Published:** 2020-04-15

**Authors:** Tadayoshi Kurita, Shingo Kawashima, Koji Morita, Yoshiki Nakajima

**Affiliations:** Department of Anesthesiology and Intensive Care, Hamamatsu University School of Medicine, 1-20-1 Handayama, Hamamatsu, 431-3192 Japan

**Keywords:** Autoregulation, Blood pressure, Cerebrum, Hypovolemia, Near-infrared spectroscopy, Spinal cord, Swine

## Abstract

**Background:**

Few studies have investigated spinal cord autoregulation using near-infrared spectroscopy (NIRS). Here, we assessed spinal cord autoregulation under normal, hypovolemic, and post-fluid resuscitation conditions compared with cerebral autoregulation.

**Methods:**

Ten pigs (36.1 ± 1.1 kg) were anesthetized with 2.5% isoflurane, before phenylephrine administration at 0.5, 1, 2, and 5 μg kg^−1^ min^−1^ in a stepwise fashion at 10-min intervals (baseline), followed by similar administration of sodium nitroprusside (SNP). Hypovolemia was induced by a 600-ml bleed (25% estimated total blood volume). Only phenylephrine was readministered (same protocol). Hypovolemia was reversed by infusing 600 ml hydroxyethyl starch, before readministering phenylephrine and SNP. The relationships between mean arterial pressure (MAP) and cerebral, thoracic, and lumbar spinal cord tissue oxygenation indices (TOIs) were evaluated.

**Results:**

Thoracic and lumbar spinal cord TOIs were approximately 15% and 10% lower, respectively, than the cerebral TOI at similar MAPs. The average relationship between MAP and each TOI showed an autoregulatory pattern, but negative correlations were observed in the cerebral TOI during phenylephrine infusion. A 600-ml bleed lowered each relationship < 5% and subsequent fluid resuscitation did not change the relationship. Individual oxygenation responses to blood pressure indicated that the spinal cord is more pressure-passive than the cerebrum. Paradoxical responses (an inverse relationship of tissue oxygenation to MAP) were observed particularly in cerebrum during phenylephrine infusion and were rare in the spinal cord.

**Conclusions:**

Spinal cord autoregulation is less robust than cerebral autoregulation and more pressure-dependent. Similar to cerebral oxygenation, spinal cord oxygenation is volume-tolerant but is more sensitive to hypotension.

## Background

The central nervous system (CNS) is thought to exhibit robust autoregulation to maintain adequate blood flow [1–3]. Recently, cerebral autoregulation was evaluated using near-infrared spectroscopy (NIRS) in several studies [4–9], which treated cerebral oxygenation as a surrogate indicator of cerebral blood flow. NIRS estimates mixed tissue hemoglobin oxygen saturation levels of the arterial, venous, and capillary blood within the field of view [10, 11], which allows the oxygen supply-and-demand balance to be evaluated under differing conditions. Similar to what has been done with the cerebrum, several studies have measured spinal cord oxygenation using NIRS directly [12–15] (via surgical procedure or thin fiber-optic probe placed in the intrathecal or epidural space) or indirectly [16–19] (via paraspinal muscle oxygenation monitoring, based on the paraspinal collateral network concept) to monitor spinal cord perfusion, but few studies to date have assessed spinal cord autoregulation using this method.

Clinical guidelines recommend elevating mean arterial pressure (MAP) to increase spinal cord perfusion in patients with acute spinal cord injury. Specifically, they recommend increasing MAP to 85–90 mmHg using vasopressors and/or intravenous fluids to protect the spinal cord and improve the neurological outcome [20]. Similarly, guidelines recommend a MAP > 60 mmHg for protecting the spinal cord during thoracoabdominal aortic surgery, because intraoperative hypotension is associated with postoperative paraplegia [21]. However, increasing blood pressure does not necessarily increase tissue oxygenation. In fact, we recently assessed cerebral and renal autoregulation using NIRS under three conditions (normal, hypovolemia following hemorrhage, and subsequent fluid resuscitation) and demonstrated that increasing blood pressure and/or fluid resuscitation after hemorrhage has less effect on cerebral oxygenation than renal oxygenation [22]. There have been few studies investigating the impact of blood pressure alterations and/or circulatory blood volume changes on spinal cord oxygenation.

We conducted the current porcine study to assess spinal cord autoregulation and cerebral autoregulation simultaneously within a single CNS and to compare the impact of MAP changes, hypovolemia, and fluid resuscitation on tissue oxygenation using NIRS.

We hypothesized that spinal cord oxygenation is more pressure-dependent than cerebral oxygenation is and that spinal cord tolerance for hypovolemia and subsequent fluid resuscitation shows minimal efficacy for improving oxygenation to a similar extent as that of the cerebrum.

## Methods

### Animal preparation

The Ethics Committee of the Animal Research Division at Hamamatsu University School of Medicine approved the study (approval number 2019025 was provided on 24 May 2019 by President Hiroyuki Konno). All experiments were performed in accordance with the Animal Research: Reporting In Vivo Experiments (ARRIVE) guidelines, with 10 swine (3 males and 7 females, mean ± SD body weight 36.1 ± 1.1 kg, range 34.5–37.9 kg, approximately 3 months old) studied from 5 June to 7 August 2019. Animals were housed with free access to food and water before the experiments. Anesthesia was induced with 5% isoflurane and oxygen using a standard animal face mask, and tracheostomy was performed. Anesthesia was maintained with 2.5% isoflurane (approximately 1.2 minimum alveolar anesthetic concentration [23]) and an oxygen–air mixture (FiO_2_ 0.6) via mechanical ventilation. Exhalation gasses were analyzed using an IntelliVue G5-M1019A (Philips Medical Systems, Eindhoven, the Netherlands). An end-tidal carbon dioxide partial pressure of 35–45 mmHg was established during the animal preparation period, and this ventilator setting was maintained throughout the study. Electrocardiographic lead II was monitored with three cutaneous electrodes. The right jugular vein was cannulated with a 14-gage double-lumen catheter and a 5F pulmonary artery catheter (Nihon Kohden, Tokyo, Japan) and the right femoral artery with a 16-gage catheter. Tracheostomy and the placement of all catheters were performed using local anesthetic (levobupivacaine or ropivacaine), under general anesthesia. Saline 100 ml h^−1^ was infused for maintenance. Body temperature (under the tongue) was maintained at 38.0–39.0 °C throughout the study with an electric heater and air conditioning.

**Fig. 1:**
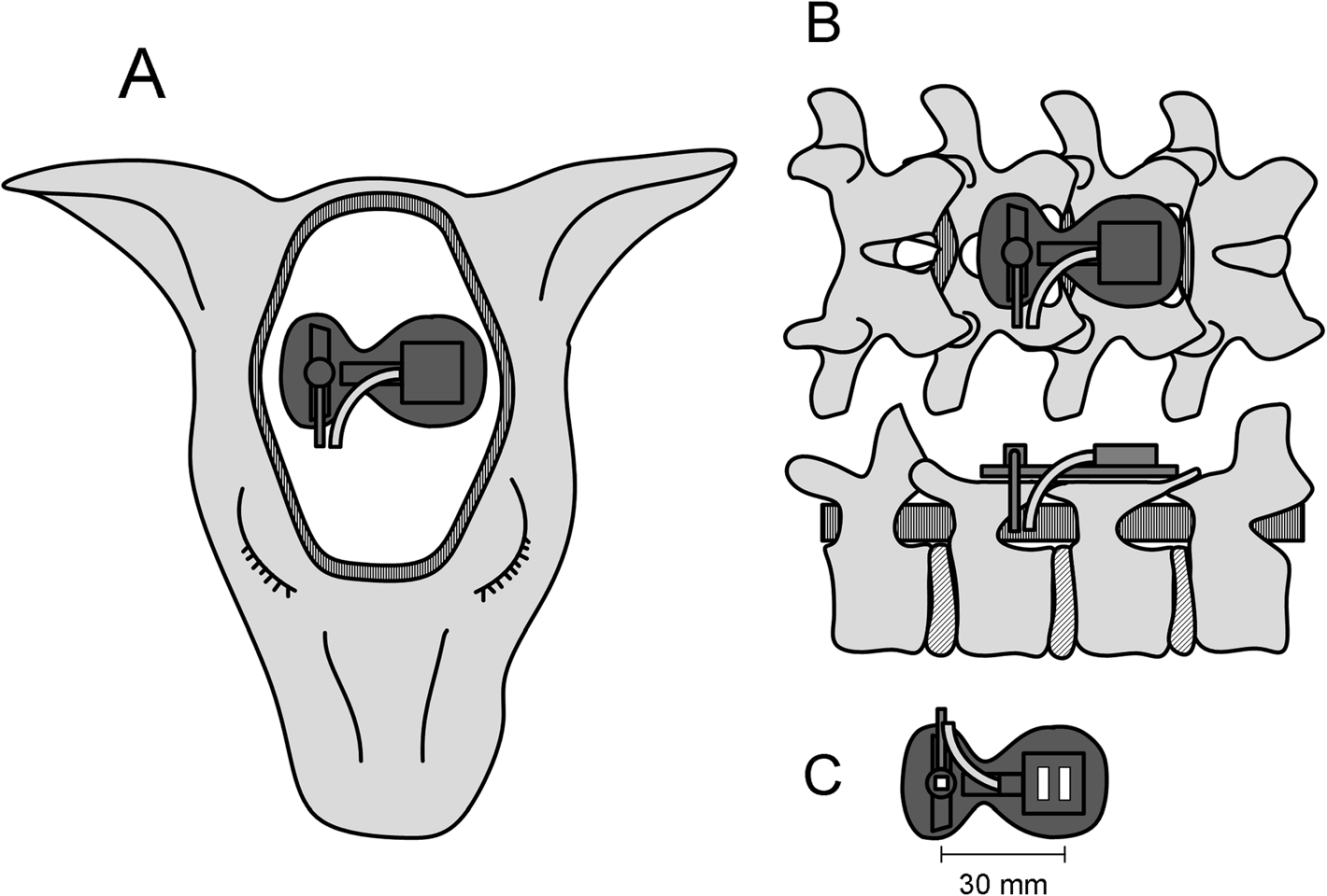
The positions of the probes, and the NIRS probe of the NIRO-200 system. A wide portion of the parietal scalp was removed, and one NIRS probe was placed on the center of the parietal skull (**a**). The other two NIRS probes were placed on the thoracic (not shown) and lumbar (**b**) laminae of the vertebral arch, which were surgically exposed and flattened after removing the spinous process. The probe consists of the emitter and receiver, housed in two aligned photodetectors, which were fixed in a rubber holder to ensure an emitter-receiver distance of 30 mm (**c**). Because the emitter-receiver distance was 30 mm, each spinal cord NIRS probe was placed on laminae approximately 20 mm from the center of the spinal cord

The pigs were placed prone. A wide portion of the parietal scalp region was removed to avoid contamination by extracranial blood [24–27], and one NIRS probe (NIRO-200; Hamamatsu Photonics, Hamamatsu, Japan) was placed on the center of the parietal skull (Fig. 1a). In the dissections performed in our pilot study [28], bone thickness in that region was 5–6 mm and in the sagittal sinus was 2–3 mm for a 35-kg pig; thus, the bones did not interfere with cerebral NIRS measurements. The other two NIRS probes were placed on the thoracic (fourth to sixth) and lumbar (first to third, Fig. 1b) laminae of the vertebral arch, which were surgically exposed and flattened after removing the spinous process, to exclude paraspinal blood flow [29]. Because we used the probe with a distance of 30 mm between the emitter and receiver (Fig. 1c), the site of the oxygenation measurement was at a depth of approximately 20 (≈ 30 × 0.7) mm. Hence, each spinal cord NIRS probe was placed on the surgically flattened lamina of the vertebral arch, approximately 20 mm from the center of the spinal cord. The distance from the surgical field to the spinal cord and the thicknesses of both the thoracic and lumbar spinal cords (approximately 10 mm) were verified in our pilot studies. We also confirmed there were no structures between the probe and the spinal cord except bone and dura mater. We used two NIRO-200 systems during the experiments because each system is equipped with only two NIRS probes. The tissue oxygenation index (TOI) was continuously measured using spatially resolved spectroscopy, with a standard procedure for the NIRO-200 system. TOI values were recorded electronically at 10-s intervals.

### Experimental protocol (Fig. 2)

After animal preparation, a 0.5 μg kg^−1^ min^−1^ phenylephrine infusion was started and increased to 1, 2, and 5 μg kg^−1^ min^−1^ at 10-min intervals and then stopped 10 min later. After 30 min, similar to the phenylephrine infusion, infusion of sodium nitroprusside (SNP) was started at 0.5 μg kg^−1^ min^−1^ and increased to 1, 2, and 5 μg kg^−1^ min^−1^ at 10-min intervals and then stopped. Heart rate (HR), MAP, mean pulmonary arterial pressure (MPA), and central venous pressure (CVP) were recorded before and after each infusion dose, and cardiac output was recorded at 0 and 5 μg kg^−1^ min^−1^. After baseline measurements, 600 ml (approximately 25% of the estimated total blood volume) of blood was removed for 15 min via the arterial catheter (hypovolemic condition). After hemodynamic parameters stabilized, a 0.5 μg kg^−1^ min^−1^ phenylephrine infusion was started and the dosage increased as described for the baseline condition. Because SNP infusion during hypovolemia induces lethal hypotension, only phenylephrine infusion was performed for the hypovolemic condition. Finally, 600 ml hydroxyethyl starch (HES) was infused for 15 min via the central venous catheter to create a post-fluid resuscitation condition. After hemodynamic parameters stabilized, phenylephrine and SNP were again infused as per the baseline condition. Arterial and mixed venous blood gasses and hematocrit were measured just before the start of the phenylephrine infusion in each condition.

**Fig. 2:**
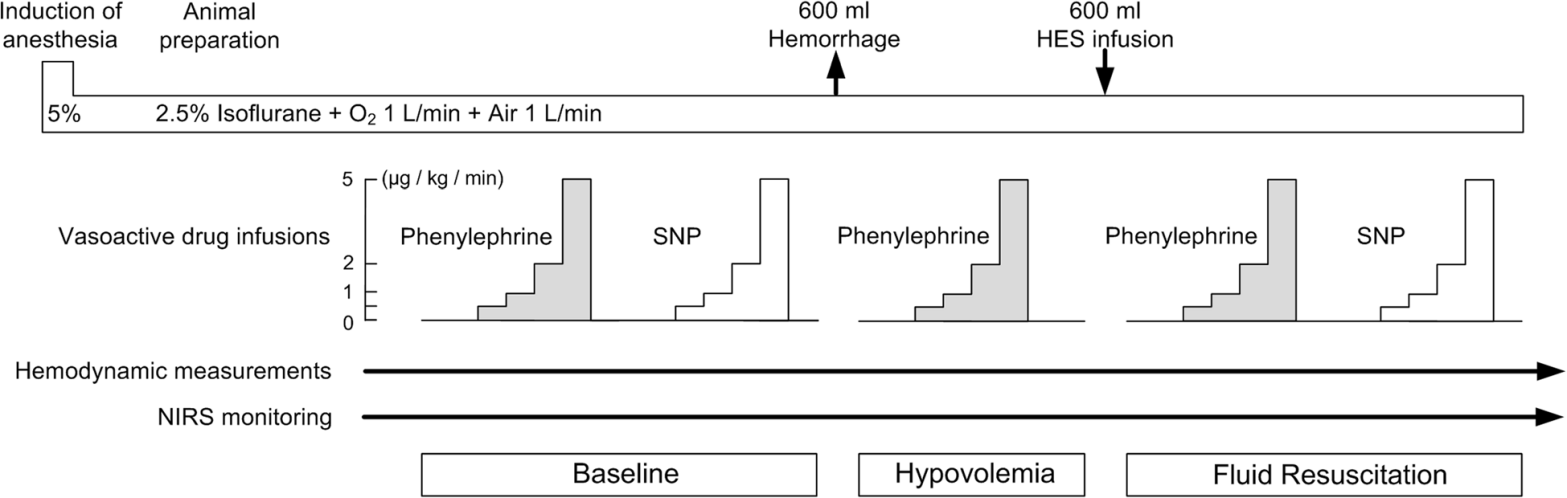
Experimental protocol. HES, hydroxyethyl starch; NIRS, near-infrared spectroscopy; SNP, sodium nitroprusside. Each animal received five stepwise infusions during the study

### Assessment of cerebral and spinal cord autoregulation

The cerebral oximetry index (COx), calculated as the Spearman’s correlation coefficient between the MAP and the cerebral TOI, was used to quantify cerebral autoregulation [4–9]. Spinal cord autoregulation was quantified in a similar fashion to the COx, by calculating the thoracic or lumbar spinal cord oximetry index (T-SOx or L-SOx, respectively). COx, T-SOx, and L-SOx were calculated by recording the values of the MAP and each TOI every 10 s for 10 min during each infusion dose, generating 60 paired samples from which the correlation coefficients between the MAP and each TOI were calculated. When autoregulation is intact, the value of each oximetry index approaches zero, whereas in a pressure-passive relationship, they approach + 1. In contrast, when there is a paradoxical response (TOI inversely related to MAP), their values are highly negative. In previous studies, the COx thresholds used to distinguish between intact and impaired autoregulation were arbitrary and were chosen by the authors as being between 0.25 and 0.5. In the present study, a threshold of 0.36 was chosen, based on a previous study from another group [4] and our swine study [22], such that we defined the following states: COx (T-SOx or L-SOx) > 0.36 (pressure-passive); 0.36 > COx (T-SOx or L-SOx) > − 0.36 (intact autoregulation); and COx (T-SOx or L-SOx) < − 0.36 (paradoxical response).

### Statistical analysis

Data are expressed as the mean ± SD. Differences in hemodynamic and arterial blood gas variables, and cerebral and spinal cord TOIs at each infusion dose and for each condition were analyzed using a repeated-measures one-way analysis of variance (ANOVA). If the ANOVA indicated significant differences, a Scheffe *F*-test for multiple comparisons was performed. Differences in COx, T-SOx, and L-SOx at each phenylephrine or SNP infusion dose, and for each condition were analyzed using the Friedman test or the Wilcoxon signed-rank test. If the Friedman test indicated a significant difference, a Steel–Dwass test for multiple comparisons was performed. Values of *P* < 0.05 were considered to indicate statistical significance.

Prior to the present study, we performed a power analysis using G*Power for Windows software (version 3.1.9.2; The G*Power Team, Universität Düsseldorf, Düsseldorf, Germany) to determine the correct sample size. We used the findings of our previous swine study [22] and calculated that at least seven animals would be required to detect significant MAP changes between baseline and phenylephrine at ≥ 1 μg kg^−1^ min^−1^ at a power of 80% and a type I error rate of 0.05 using a two-sided *t* test.

## Results

All pigs (*n* = 10) were kept alive throughout the experiment. Because the data for the lumbar spinal cord TOIs were lost from one pig, the lumbar spinal cord results were evaluated from nine animals. Phenylephrine dose-dependently decreased the HR and increased the MAP, MPA, and CVP across all conditions, and SNP dose-dependently increased the HR and decreased the MAP, MPA, and CVP (the hemodynamic and blood gas variables during the experiments are shown in additional file 1). A 600-ml bleed increased HR and decreased MAP, MPA, CVP, and cardiac output, and subsequent fluid resuscitation decreased HR and increased MPA, CVP, and cardiac output above the baseline condition. Arterial carbon dioxide partial pressure (PaCO_2_) before the start of the phenylephrine infusion did not differ significantly between conditions (*P* = 0.13; 42 ± 3, 44 ± 4, and 42 ± 3 mmHg, respectively).

Table 1 shows the MAP and TOI values during the experiments. The cerebral TOI values were stable, despite drastic MAP changes, with only SNP significantly decreasing the cerebral TOI during the baseline condition and after fluid resuscitation (5%, *P* = 0.0008; and 9%, *P* < 0.0001 vs. pre-infusion, respectively). In contrast, spinal cord oxygenation was more sensitive to pressure changes. The thoracic spinal cord TOI significantly increased under the baseline condition (3%, *P* = 0.0051 vs. pre-infusion), and the thoracic and lumbar spinal cord TOIs increased during the hypovolemic condition (10%, *P* < 0.0001; and 11%, *P* = 0.0003 vs. pre-infusion, respectively) during phenylephrine infusions. The thoracic and lumbar spinal cord TOIs decreased under the baseline (11%, *P* < 0.0001; and 10%, *P* < 0.0001 vs. pre-infusion, respectively) and post-fluid resuscitation conditions (13%, *P* < 0.0001; and 15%, *P* < 0.0001 vs. pre-infusion, respectively) during SNP infusions. The 600-ml bleed decreased the cerebral, thoracic, and lumbar spinal cord TOIs (6%, *P* = 0.0051; 12%, *P* = 0.0008; and 13%, *P* = 0.0033 vs. the baseline condition, respectively), whereas only the thoracic spinal cord TOI changed significantly after fluid resuscitation (*P* = 0.0258 vs. the hypovolemic condition). Hypotension and hemorrhage decreased spinal cord TOIs approximately twofold compared with the cerebral TOI. Both spinal cord TOIs were significantly lower (by approximately 10–15%) than the cerebral TOI during each vasoactive drug infusion dose, in each condition (all *P* values in the range from 0.0001 to 0.0003).

**Table 1:** Mean arterial pressure (MAP) and each tissue oxygenation index (TOI) during experiments

Baseline					
Phenylephrine dose	0 μg kg^−1^ min^−1^	0.5 μg kg^−1^ min^−1^	1 μg kg^−1^ min^−1^	2 μg kg^−1^ min^−1^	5 μg kg^−1^ min^−1^
MAP (mmHg)	60 ± 3	64 ± 3	69 ± 4*	80 ± 7^‡^	109 ± 14^#^
Cerebral TOI (%)	64.9 ± 2.6	64.5 ± 2.9	64.2 ± 4.0	63.4 ± 4.3	62.5 ± 4.3
T-spinal cord TOI (%)	47.7 ± 3.7	47.6 ± 3.8	47.6 ± 3.7	48.4 ± 3.8	49.2 ± 3.7^‡^
L-spinal cord TOI (%)	52.8 ± 2.8	53.3 ± 2.7	53.9 ± 3.3	54.6 ± 3.9	54.9 ± 4.6
SNP dose	0 μg kg^−1^ min^−1^	0.5 μg kg^−1^ min^−1^	1 μg kg^−1^ min^−1^	2 μg kg^−1^ min^−1^	5 μg kg^−1^ min^−1^
MAP (mmHg)	69 ± 8	59 ± 7*	54 ± 6*	49 ± 7^†^	41 ± 7^#^
Cerebral TOI (%)	63.9 ± 3.4	63.2 ± 2.8	63.1 ± 2.4	62.4 ± 2.2	60.8 ± 2.6^‡^
T-spinal cord TOI (%)	47.2 ± 3.7	45.7 ± 3.2	45.1 ± 2.9*	43.9 ± 2.9*	41.8 ± 3.1^#^
L-spinal cord TOI (%)	53.2 ± 4.0	51.8 ± 3.7	51.6 ± 4.3	50.4 ± 4.3*	47.7 ± 4.0^#^
Hypovolemia					
Phenylephrine dose	0 μg kg^−1^ min^−1^	0.5 μg kg^−1^ min^−1^	1 μg kg^−1^ min^−1^	2 μg kg^−1^ min^−1^	5 μg kg^−1^ min^−1^
MAP (mmHg)	48 ± 8	53 ± 7	56 ± 6*	60 ± 8^†^	80 ± 11^#^
Cerebral TOI (%)	61.2 ± 3.1	61.4 ± 2.8	61.2 ± 2.8	60.3 ± 2.3	60.2 ± 2.3
T-spinal cord TOI (%)	42.2 ± 4.7	43.4 ± 4.5	44.4 ± 3.9*	45.1 ± 3.4*	46.5 ± 3.6^‡^
L-spinal cord TOI (%)	46.2 ± 3.6	47.6 ± 3.8	49.0 ± 4.3	49.7 ± 4.4*	51.1 ± 4.8^†^
Fluid resuscitation					
Phenylephrine dose	0 μg kg^−1^ min^−1^	0.5 μg kg^−1^ min^−1^	1 μg kg^−1^ min^−1^	2 μg kg^−1^ min^−1^	5 μg kg^−1^ min^−1^
MAP (mmHg)	60 ± 7	61 ± 6	64 ± 8	75 ± 10^‡^	102 ± 21^#^
Cerebral TOI (%)	61.0 ± 3.5	60.8 ± 3.1	60.6 ± 2.9	60.3 ± 2.2	59.4 ± 2.0
T-spinal cord TOI (%)	45.7 ± 4.2	45.1 ± 3.9	45.1 ± 3.5	45.9 ± 3.5	46.2 ± 3.8
L-spinal cord TOI (%)	50.1 ± 4.3	49.4 ± 4.0	49.4 ± 3.8	50.3 ± 4.5	50.7 ± 5.1
SNP dose	0 μg kg^−1^ min^−1^	0.5 μg kg^−1^ min^−1^	1 μg kg^−1^ min^−1^	2 μg kg^−1^ min^−1^	5 μg kg^−1^ min^−1^
MAP (mmHg)	56 ± 7	48 ± 5*	44 ± 5*	39 ± 5^‡^	34 ± 3^#^
Cerebral TOI (%)	60.2 ± 3.0	59.0 ± 2.4	58.8 ± 3.2	57.5 ± 2.6*	54.7 ± 3.0^#^
T-spinal cord TOI (%)	43.9 ± 2.9	41.9 ± 2.7*	41.6 ± 2.5*	40.4 ± 2.7^†^	38.4 ± 2.6^#^
L-spinal cord TOI (%)	48.1 ± 4.0	46.0 ± 3.9	45.7 ± 4.8	44.2 ± 4.6*	41.1 ± 3.2^#^

Data are expressed as the mean ± SD. *L-spinal cord TOI* lumbar spinal cord TOI, *SNP* sodium nitroprusside, *T-spinal cord TOI* thoracic spinal cord TOI

*P < 0.05 versus period prior to phenylephrine or SNP infusion (0 μg kg^−1^ min^−1^); ^†^*P* < 0.05 versus 0 and 0.5 μg kg^−1^ min^−1^; ^‡^*P* < 0.05 versus 0, 0.5, and 1 μg kg^−1^ min^−1^; ^#^*P* < 0.05 versus all other infusion doses

**Fig. 3:**
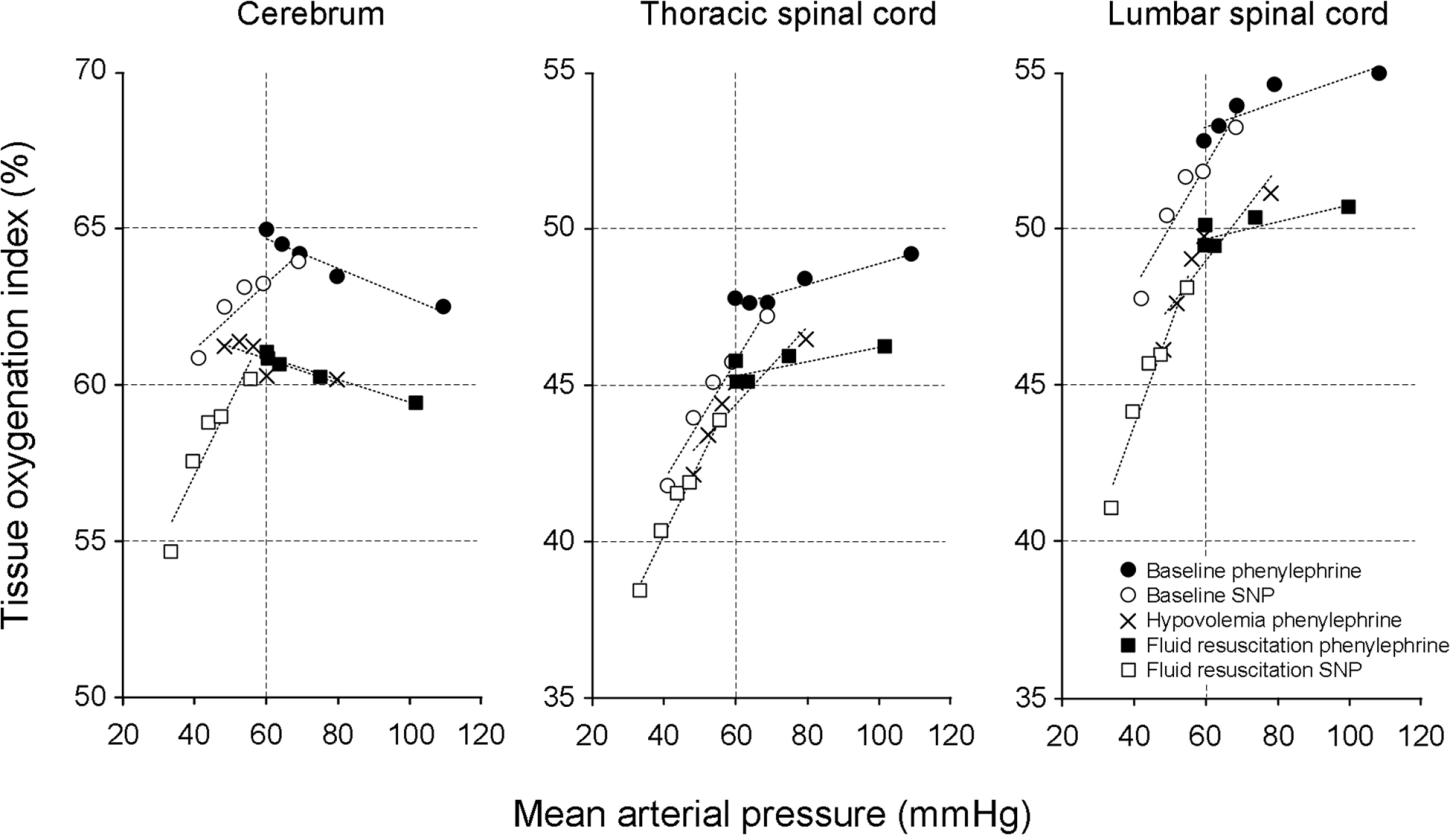
Relationship between the mean arterial pressure and each tissue oxygenation index. The linear regression lines for the cerebrum, thoracic spinal cord, and lumbar spinal cord data, respectively, are *y* = − 0.05*x* + 68 (*R*^2^ = 0.95), *y* = 0.03*x* + 46 (*R*^2^ = 0.94), and *y* = 0.04*x* + 51 (*R*^2^ = 0.78) during phenylephrine infusion under baseline conditions; *y* = 0.10*x* + 57 (*R*^2^ = 0.89), *y* = 0.19*x* + 34 (*R*^2^ = 0.97), and *y* = 0.20*x* + 40 (*R*^2^ = 0.91) during sodium nitroprusside (SNP) infusion under baseline conditions; *y* = − 0.04*x* + 63 (*R*^2^ = 0.67), *y* = 0.12*x* + 37 (*R*^2^ = 0.87), and *y* = 0.15*x* + 40 (*R*^2^ = 0.82) during phenylephrine infusion under hypovolemia; *y* = − 0.04*x* + 63 (*R*^2^ = 0.97), *y* = 0.02*x* + 44 (*R*^2^ = 0.60), and *y* = 0.03*x* + 48 (*R*^2^ = 0.65) during phenylephrine infusion under fluid resuscitation; and *y* = 0.24*x* + 48 (*R*^2^ = 0.88), *y* = 0.24*x* + 31 (*R*^2^ = 0.98), and *y* = 0.32*x* + 31 (*R*^2^ = 0.95) during SNP infusion under fluid resuscitation

Figure 3 shows the relationship between MAP and each of the TOI measurements. The cerebral TOI during phenylephrine infusion showed a negative correlation in all conditions, whereas spinal cord TOIs showed positive correlations. The relationships shifted less than 5% downward after the 600-ml bleed for both the cerebral and spinal cord TOIs and showed no change after 600-ml HES infusion.

Paradoxical responses were observed in the cerebral TOI of all 10 animals during any stepwise infusion, in the thoracic spinal cord TOI in 6 of the 10 animals, and in the lumbar spinal cord TOI in 6 of the 9 animals. However, the total number of stepwise infusions during which paradoxical responses were observed across animals was markedly lower for the thoracic and lumbar spinal cord TOIs than for the cerebral TOIs (10 of 50 and 8 of 45 vs. 21 of 50 stepwise infusions, respectively).

The median values of COx, T-SOx, and L-SOx, and the response patterns recorded are shown in Table 2. During phenylephrine infusion, COx decreased with increasing infusion dose, and the number of paradoxical responses increased instead of decreasing, as in the pressure-passive response; however, this phenomenon was not clear in both spinal cord oximetry indices. Pressure-passive responses were more frequent in the spinal cord, and paradoxical responses were less frequent in the spinal cord than in the brain. During SNP infusion, the majority of responses in both organs were pressure-passive (oxygenation decreased with decreasing MAP). Paradoxical reactions were rarely observed, with only two cases observed in the thoracic spinal cord after fluid resuscitation, one in the lumbar spinal cord at baseline, and no occurrences in the cerebrum.

**Table 2:** Median values and ranges of each oximetry index and response pattern counts

Cerebral oximetry index (*n* = 10)					
Dose	Baseline		Hypovolemia	Fluid resuscitation	
	Phenylephrine	SNP	Phenylephrine	Phenylephrine	SNP
0.5	0.05 (− 0.83 to 0.89)	0.76 (0.10 to 0.97)	0.36 (− 0.30 to 0.77)	0.15 (− 0.51 to 0.85)	0.77 (− 0.28 to 0.98)
1	− 0.41 (− 0.81 to 0.86)	0.27 (− 0.20 to 0.97)	0.27 (− 0.19 to 0.76)^ǁ^	0.10 (− 0.42 to 0.67)	0.70 (0.03 to 0.96)
2	− 0.21 (− 0.88 to 0.63)	0.81 (0.34 to 0.96)	0.12 (− 0.76 to 0.86)	− 0.03 (− 0.88 to 0.72)	0.91 (0.31 to 0.96)
5	− 0.66 (− 0.93 to 0.92)	0.93 (0.00 to 0.98)^†^	− 0.31 (− 0.87 to 0.71)	− 0.31 (− 0.85 to 0.79)	0.95 (0.87 to 0.98)^†^
Response pattern counts (Pressure-passive/intact autoregulation/paradoxical response)					
0.5	3/4/3	7/3/0	5/5/0	4/5/1	8/2/0
1	1/3/6	4/6/0	4/6/0	3/5/2	7/3/0
2	1/5/4	9/1/0	3/3/4	1/6/3	9/1/0
5	1/2/7	9/1/0	2/3/5	1/5/4	10/0/0
Thoracic spinal cord oximetry index (*n* = 10)					
Dose	Baseline		Hypovolemia	Fluid resuscitation	
	Phenylephrine	SNP	Phenylephrine	Phenylephrine	SNP
0.5	0.44 (− 0.87 to 0.91)	0.89 (0.76 to 0.96)	0.75 (0.00 to 0.93)	0.12 (− 0.82 to 0.79)	0.89 (0.47 to 0.94)
1	0.49 (− 0.85 to 0.86)	0.80 (− 0.32 to 0.93)	0.44 (− 0.24 to 0.92)	0.25 (− 0.72 to 0.57)	0.72 (− 0.50 to 0.89)
2	0.57 (− 0.78 to 0.96)	0.82 (0.25 to 0.89)	0.78 (− 0.38 to 0.91)	0.55 (− 0.64 to 0.87)	0.83 (− 0.41 to 0.90)
5	0.29 (− 0.57 to 0.82)	0.80 (0.23 to 0.93)	0.53 (0.06 to 0.87)	− 0.11 (− 0.85 to 0.60)	0.84 (0.57 to 0.96)
Response pattern counts (Pressure-passive/intact autoregulation/paradoxical response)					
0.5	6/3/1	10/0/0	8/2/0	4/4/2	10/0/0
1	5/2/3	8/2/0	6/4/0	2/6/2	9/0/1
2	7/1/2	9/1/0	7/2/1	7/2/1	9/0/1
5	5/4/1	9/1/0	6/4/0	3/3/4	10/0/0
Lumbar spinal cord oximetry index (*n* = 9)					
Dose	Baseline		Hypovolemia	Fluid resuscitation	
	Phenylephrine	SNP	Phenylephrine	Phenylephrine	SNP
0.5	0.46 (− 0.88 to 0.92)	0.86 (0.51 to 0.96)	0.59 (0.12 to 0.80)	− 0.04 (− 0.19 to 0.68)	0.87 (0.44 to 0.93)
1	0.22 (− 0.87 to 0.83)	0.45 (− 0.47 to 0.88)*	0.46 (− 0.32 to 0.92)	0.00 (− 0.34 to 0.58)	0.68 (0.31 to 0.84)
2	0.45 (− 0.90 to 0.97)	0.80 (0.54 to 0.89)	0.68 (− 0.39 to 0.92)	0.45 (− 0.87 to 0.91)	0.87 (0.61 to 0.94)
5	− 0.11 (− 0.99 to 0.63)	0.75 (0.53 to 0.85)	0.36 (− 0.33 to 0.97)	0.02 (− 0.87 to 0.43)	0.85 (0.65 to 0.97)
Response pattern counts (pressure-passive/intact autoregulation/paradoxical response)					
0.5	5/2/2	9/0/0	6/3/0	4/5/0	9/0/0
1	4/2/3	5/3/1	5/4/0	2/7/0	8/1/0
2	7/1/1	9/0/0	6/2/1	5/3/1	9/0/0
5	2/5/2	9/0/0	5/4/0	2/4/3	9/0/0

*Dose* each vasoactive drug infusion dose (μg kg^−1^ min^−1^), *SNP* sodium nitroprusside. Response patterns were defined as follows: an oximetry index > 0.36 was defined as pressure-passive, an oximetry index between − 0.36 and 0.36 was defined as intact autoregulation, and an oximetry index < − 0.36 was defined as a paradoxical response

*P < 0.05 versus 0.5 μg kg^−1^ min^−1^; ^†^*P* < 0.05 versus 1 μg kg^−1^ min^−1^; ^ǁ^*P* < 0.05 versus the same dose of phenylephrine infusion in the baseline condition

## Discussion

The present study investigated spinal cord autoregulation compared with cerebral autoregulation using NIRS under normal, hemorrhage-induced hypovolemic, and subsequent fluid resuscitation conditions, which are common scenarios in perioperative and/or critical care patients. Our findings indicate that thoracic and lumbar spinal cord TOIs showed similar changes throughout the experiments, with these TOIs being approximately 10–15% lower than the cerebral TOI at similar MAPs. Hypotension and hemorrhage decreased spinal cord TOIs twofold compared with the cerebral TOI, and fluid resuscitation had minimal impact in terms of increasing any of the TOIs. Individual responses indicated that spinal cord oxygenation is more pressure-passive than cerebral oxygenation. The most paradoxical responses were observed in the cerebrum during vasopressor infusion, similar to those found in our previous animal study [22], but were rare in spinal cord oxygenations.

### Cerebral and spinal cord oxygenation in response to blood pressure changes

The changes in oxygenation in response to blood pressure alterations were different between the cerebrum and spinal cord. Cerebral oxygenation was pressure-tolerant, but the relationship between the MAP and cerebral TOI did not fit the classic autoregulation shape, because negative correlations were observed during phenylephrine infusions. These findings suggest that negative TOI responses were more frequent than positive responses during phenylephrine infusions. Our results also indicate that the cerebral TOI at a MAP of 49 mmHg (62.4%) under the baseline condition (during 2 μg kg^−1^ min^−1^ of SNP infusion) was higher than all other TOI values in the subsequent experiments, suggesting that hypotension might be tolerated by the cerebrum in the presence of a normal hemoglobin concentration. In contrast, the spinal cord TOIs changed in a more pressure-dependent manner. Pressure-passive responses were observed more frequently than in cerebrum, and spinal cord TOIs decreased twofold during SNP infusion. We note that the effect of phenylephrine on spinal cord TOIs differed depending on the condition and that these TOIs were increased most effectively during hypovolemia (Table 1). One might speculate that the spinal cord perfusion pressure increased because of the decrease in CVP (outflow pressure) during hypovolemia. However, similar TOIs were shown for the hypovolemia and fluid resuscitation conditions at a similar MAP (Fig. 3), indicating that the effect of phenylephrine during hypovolemia reflected baseline blood pressure. Our findings indicate that spinal cord oxygenation is pressure-dependent regardless of the systemic volume.

### The impact of hypovolemia and subsequent fluid resuscitation on oxygenation

The 600-ml bleed decreased spinal cord TOIs twofold more than it decreased the cerebral TOI, reflecting the impact of the decrease in blood pressure on each TOI. However, the relationship between the MAP and each TOI shifted less than 5% downward and fluid resuscitation did not change the relationship with each TOI. These findings indicate that CNS is volume-tolerant and that hypovolemia induces circulatory blood redistribution to the CNS, with the reduction in TOIs being limited. In fact, as we demonstrated in our previous study, renal oxygenation drastically changes depending on volume conditions, with the relationship between the MAP and renal TOI decreasing fourfold compared with the cerebral TOI, because of hypovolemia (the renal TOI decreased from 62 to 42% at 60 mmHg MAP). Subsequent fluid resuscitation reduced this to a twofold decrease (the renal TOI recovered from 42 to 52% at 60 mmHg MAP), despite the cerebral TOI showing similar changes to the present study [22]. We speculate that fluid resuscitation contributes more to the other organs compared with the organs of the CNS and that it might be only minimally effective at increasing spinal cord oxygenation. Based on the relationship between the MAP and each TOI (Fig. 3), we also speculate that blood transfusion might be more effective than fluid resuscitation and/or elevating blood pressure, but additional studies are required to confirm this possibility.

### Paradoxical oxygenation responses to blood pressure changes

An inverse change in TOI, relative to blood pressure, does not fit the classic autoregulation concept but is commonly observed in animals [22] and in clinical patients [7]. In the present study, paradoxical responses were observed in the cerebral TOI in all animals during any stepwise infusions and occurred frequently with an increasing dose of phenylephrine. Although some previous reports have demonstrated that a phenylephrine bolus decreases the cerebral NIRS signal because of a decrease in cardiac output [30, 31], our findings indicate that paradoxical responses did not occur secondary to a decrease in cardiac output (additional file 1). As a part of the normal physiological response that prevents excessive increases in cerebral blood flow [7, 22], the rapid changes in perfusion pressure induce arteriolar vasoconstriction as the pressure increases and arteriolar vasodilation as the pressure decreases. The resulting change in the arterial-to-venous blood volume ratio explains the paradoxical response [32]. In our previous study, animals with paradoxical responses in their cerebral TOIs (60% of animals) maintained more stable cerebral oxygenation levels than those that did not show a paradoxical response, suggesting that this response is preferable to autoregulation for controlling appropriate blood flow [22]. The spinal cord has a similar regulatory process, but it may not be as robust as that of the cerebrum. During SNP infusions, paradoxical reactions were observed twice in the thoracic spinal cord, once in the lumbar spinal cord, and never in the cerebrum, indicating that the effectiveness of increasing oxygenation during decreasing blood pressure might be low and should not be expected to help in this case.

### Study limitations

Several limitations of the present study should be addressed. We did not administer phenylephrine and SNP in a crossover design, with each stage occurring sequentially. It is possible, therefore, that the drug infusion order and/or repetitive drug administration during autoregulation assessment could have influenced the subsequent measurements. We induced a wide range of MAPs using phenylephrine and SNP under isoflurane (cerebral vasodilator) anesthesia. However, the results may differ if other vasoactive drugs are used to alter the MAP, either under anesthesia induced by other drugs or without anesthesia. β1 stimulants [33] and norepinephrine [34] in particular can increase cerebral and spinal cord oxygenation compared with phenylephrine. Furthermore, although we did not measure blood gasses during vasoactive drug infusions, it is possible that PaCO_2_ changed with hemodynamic changes and affected the autoregulation assessment. We speculate, however, that PaCO_2_ was not likely to have changed much because cardiac output did not significantly change, even after 5 μg kg^−1^ min^−1^ of vasoactive drug infusions, except during phenylephrine infusion after fluid resuscitation (additional file 1). In addition, in the current study, autoregulation was evaluated using only oximetry indices, which were derived straightforwardly from the Spearman’s correlation coefficient between the MAP and each TOI. Additionally, the correlation coefficient threshold that was chosen to distinguish between intact and impaired autoregulation was set at 0.36 based on a previous study [4], but this was chosen arbitrarily. Finally, the present study used NIRS as a surrogate indicator of blood flow, similar to previous studies [4–9], but NIRS evaluates the oxygen supply-and-demand balance by measuring mixed tissue hemoglobin oxygen saturation within the optical field of view. The alteration of blood flow likely changes the TOI, but a given blood flow change does not necessarily cause the same TOI change under conditions where the oxygen supply-and-demand and/or regional blood volume change. In the present study, the oxygen delivery obviously differed between the three conditions. Furthermore, the regional metabolism and/or regional blood volume might have differed during the experiment, due to several interventions (vasoactive drug infusion, hemorrhage, and fluid resuscitation). These findings indicate that the effects of the changes in blood flow on the TOI did not remain constant during the experiment, suggesting that TOI might not accurately reflect blood flow. This might be an unavoidable limitation when NIRS is used as a surrogate indicator of blood flow.

## Conclusions

Although our results cannot be directly extrapolated to humans and further clinical studies are required to validate our results, they show that spinal cord autoregulation is not as robust as cerebral autoregulation and that oxygenation in the spinal cord is more pressure-dependent. The paradoxical oxygenation response to blood pressure was rare in the spinal cord in contrast to the brain. While spinal cord oxygenation is volume-tolerant similar to cerebral oxygenation, avoiding low blood pressure is more important for maintaining the oxygenation level in the spinal cord.

## Supplementary information

**Additional file 1.** Blood gases and hemodynamic variables during experiments.

## Data Availability

The datasets used and/or analyzed during the current study are available from the corresponding author on reasonable request.

## Abbreviations

CNS: Central nervous system
COx: Cerebral oximetry index
CVP: Central venous pressure
HES: Hydroxyethyl starch
HR: Heart rate
L-SOx: Lumbar spinal cord oximetry index
MAP: Mean arterial pressure
MPA: Mean pulmonary arterial pressure
NIRS: Near-infrared spectroscopy
PaCO_2_: Arterial carbon dioxide partial pressure
SNP: Sodium nitroprusside
TOI: Tissue oxygenation index
T-SOx: Thoracic spinal cord oximetry index
