# Supplementary material for: Spinal cord autoregulation using near-infrared spectroscopy under normal, hypovolemic, and post-fluid resuscitation conditions in a swine model: a comparison with cerebral autoregulation
Source: J Intensive Care. 2020 Apr 15;8:27. doi: 10.1186/s40560-020-00443-6 (PMC7158138; doi:10.1186/s40560-020-00443-6)
Supplement: Supplementary file 1 — Additional file 1. Blood gases and hemodynamic variables during experiments. [file 40560_2020_443_MOESM1_ESM.docx]

Additional file 1. Blood gases and hemodynamic variables during experiments.

| Baseline | | | | | |
| --- | --- | --- | --- | --- | --- |
| Phenylephrine  dose | 0  µg kg^-1^ min^-1^ | 0.5  µg kg^-1^ min^-1^ | 1  µg kg^-1^ min^-1^ | 2  µg kg^-1^ min^-1^ | 5  µg kg^-1^ min^-1^ |
| pH | 7.48 ± 0.03 | ̶̶ | ̶ | ̶ | ̶ |
| PaO_2_ (mmHg) | 232 ± 22 | ̶̶ | ̶ | ̶ | ̶ |
| PaCO_2_ (mmHg) | 42 ± 3 | ̶̶ | ̶ | ̶ | ̶ |
| SvO_2_ (%) | 72 ± 6 | ̶̶ | ̶ | ̶ | ̶ |
| Hematocrit (%) | 33 ± 3 | ̶̶ | ̶ | ̶ | ̶ |
| HR (beats min^-1^) | 132 ± 26 | 120 ± 24 | 111 ± 20 * | 101 ±14 † | 105 ± 15 * |
| MAP (mmHg) | 60 ± 3 | 64 ± 3 | 69 ± 4 * | 80 ± 7 ‡ | 109 ± 14 # |
| MPA (mmHg) | 16 ± 2 | 17 ± 2 | 18 ± 2 * | 20 ± 1 ‡ | 25 ± 2 # |
| CVP (mmHg) | 6 ± 3 | 8 ± 3 | 9 ± 4 * | 10 ± 4 † | 13 ± 5 # |
| CO (L min^-1^) | 3.4 ± 0.7 | ̶̶ | ̶ | ̶ | 3.3 ± 0.8 |
| SNP  dose | 0  µg kg^-1^ min^-1^ | 0.5  µg kg^-1^ min^-1^ | 1  µg kg^-1^ min^-1^ | 2  µg kg^-1^ min^-1^ | 5  µg kg^-1^ min^-1^ |
| HR (beats min^-1^) | 115 ± 28 | 118 ± 25 | 118 ± 20 | 117 ± 22 | 123 ± 24 |
| MAP (mmHg) | 69 ± 8 | 59 ± 7 * | 54 ± 6 * | 49 ± 7 † | 41 ± 7 # |
| MPA (mmHg) | 16 ± 2 | 14 ± 2 * | 13 ± 2 † | 12 ± 2 † | 11 ± 2 ‡ |
| CVP (mmHg) | 6 ± 4 | 5 ± 3 | 4 ± 3 * | 4 ± 3 † | 2 ± 3 ‡ |
| CO (L min^-1^) | 3.2 ± 0.5 | ̶ | ̶ | ̶ | 2.8 ± 0.7 |
| Hypovolemia | | | | | |
| Phenylephrine  dose | 0  µg kg^-1^ min^-1^ | 0.5  µg kg^-1^ min^-1^ | 1  µg kg^-1^ min^-1^ | 2  µg kg^-1^ min^-1^ | 5  µg kg^-1^ min^-1^ |
| pH | 7.42 ± 0.04 | ̶̶ | ̶ | ̶ | ̶ |
| PaO_2_ (mmHg) | 155 ± 19 | ̶̶ | ̶ | ̶ | ̶ |
| PaCO_2_ (mmHg) | 44 ± 4 | ̶̶ | ̶ | ̶ | ̶ |
| SvO_2_ (%) | 52 ± 12 | ̶̶ | ̶ | ̶ | ̶ |
| Hematocrit (%) | 30 ± 4 | ̶̶ | ̶ | ̶ | ̶ |
| HR (beats min^-1^) | 164 ± 31 | 162 ± 31 | 153 ± 29 | 133 ± 21 ‡ | 118 ± 19 ‡ |
| MAP (mmHg) | 48 ± 8 | 53 ± 7 | 56 ± 6 * | 60 ± 8 † | 80 ± 11 # |
| MPA (mmHg) | 14 ± 2 | 14 ± 2 | 15 ± 2 | 17 ± 2 † | 21 ± 2 # |
| CVP (mmHg) | 2 ± 3 | 3 ± 3 | 3 ± 4 | 4 ± 4 | 6 ± 4 ‡ |
| CO (L min^-1^) | 2.4 ± 0.5 | ̶ | ̶ | ̶ | 2.5 ± 0.5 |
| Fluid resuscitation | | | | | |
| Phenylephrine  dose | 0  µg kg^-1^ min^-1^ | 0.5  µg kg^-1^ min^-1^ | 1  µg kg^-1^ min^-1^ | 2  µg kg^-1^ min^-1^ | 5  µg kg^-1^ min^-1^ |
| pH | 7.45 ± 0.03 | ̶̶ | ̶ | ̶ | ̶ |
| PaO_2_ (mmHg) | 187 ± 15 | ̶̶ | ̶ | ̶ | ̶ |
| PaCO_2_ (mmHg) | 42 ± 3 | ̶̶ | ̶ | ̶ | ̶ |
| SvO_2_ (%) | 68 ± 10 | ̶̶ | ̶ | ̶ | ̶ |
| Hematocrit (%) | 19 ± 3 | ̶̶ | ̶ | ̶ | ̶ |
| HR (beats min^-1^) | 114 ± 14 | 110 ± 12 | 105 ± 12 * | 99 ± 12 ‡ | 101 ± 10 † |
| MAP (mmHg) | 60 ± 7 | 61 ± 6 | 64 ± 8 | 75 ± 10 ‡ | 102 ± 21 # |
| MPA (mmHg) | 19 ± 2 | 18 ± 2 | 18 ± 2 | 20 ± 2 | 24 ± 3 # |
| CVP (mmHg) | 9 ± 3 | 9 ± 3 | 9 ± 3 | 11 ± 3 * | 14 ± 4 # |
| CO (L min^-1^) | 3.8 ± 0.4 | ̶ | ̶ | ̶ | 3.1 ± 0.7 * |
| SNP  dose | 0  µg kg^-1^ min^-1^ | 0.5  µg kg^-1^ min^-1^ | 1  µg kg^-1^ min^-1^ | 2  µg kg^-1^ min^-1^ | 5  µg kg^-1^ min^-1^ |
| HR (beats min^-1^) | 103 ± 14 | 110 ± 17 | 115 ± 18 | 120 ± 20 * | 129 ± 23 ‡ |
| MAP (mmHg) | 56 ± 7 | 48 ± 5 * | 44 ± 5 * | 39 ± 5 ‡ | 34 ± 3 # |
| MPA (mmHg) | 15 ± 2 | 12 ± 3 * | 12 ± 2 * | 11 ± 2 † | 10 ± 3 ‡ |
| CVP (mmHg) | 6 ± 3 | 4 ± 3 * | 4 ± 4 * | 3 ± 3 * | 2 ± 4 † |
| CO (L min^-1^) | 3.1 ± 0.4 | ̶ | ̶ | ̶ | 2.9 ± 0.5 |

Data are expressed as the mean ± SD. Blood gases were measured only at 0 µg kg^-1^ min^-1^ of phenylephrine (just before the start of phenylephrine infusion) and CO was measured only at 0 and 5 µg kg^-1^ min^-1^ of each drug infusion. CO, cardiac output; CVP, central venous pressure; HR, heart rate; MAP, mean arterial pressure; MPA, mean pulmonary arterial pressure; PaCO_2_, arterial carbon dioxide partial pressure; PaO_2_, arterial oxygen partial pressure; SNP, sodium nitroprusside; SvO_2_, mixed venous oxygen saturation.

* P < 0.05 versus period prior to phenylephrine or SNP infusion (0 µg kg^-1^ min^-1^), † P < 0.05 versus 0 and 0.5 µg kg^-1^ min^-1^, ‡ P < 0.05 versus 0, 0.5, and 1 µg kg^-1^ min^-1^, # P < 0.05 versus all other infusion doses.
